# *Taenia solium* development and host interactions in neurocysticercosis: a narrative mini review on mechanistic pathways to epileptogenesis

**DOI:** 10.1186/s42494-026-00261-7

**Published:** 2026-06-03

**Authors:** Franck Katembo Sikakulya, Hervé Monka Lekuya, Larry Kasereka Kamabu, Mathieu Katembo Manzekele, Eric Ochen, Fatuma Djuma Sonia, Jeannot Munihire Baanitse, Furaha Nzanzu Blaise Pascal, Rishi A. Shah, Shitu Hauwa Balarabe, Olivier Kambere Kavulikirwa, David Kitya, Malcolm K. Jones

**Affiliations:** 1 Neurosurgery Division, Faculty of Medicine, Université Catholique du Graben, P.O. Box 29, Butembo, DR Congo; 2 Neurosurgery Division, Mbarara Regional Referral Hospital, P.O. Box 40, Mbarara, Uganda; 3 Department of Surgery, College of Health Sciences, Makerere University, P.O. Box 7062, Kampala, Uganda; 4 Department of Human Repair, Neurosurgery, Ghent University, P.O. Box 9000, Ghent, Belgium; 5 Department of Public Health, Mbarara University of Science and Technology, P.O. Box 1410, Mbarara, Uganda; 6 Department of Surgery, Faculty of Clinical Medicine and Dentistry, Kampala International University Western Campus, P.O. Box 71, Bushenyi, Uganda; 7 Department of Anaesthesia and Critical Care, Faculty of Medicine, Université Catholique du Graben, P.O. Box 29, Butembo, DR Congo; 8 Department of Global Public Health, University of Virginia, P.O. Box 400183, Charlottesville, VA USA; 9 Faculty of Veterinary Medicine, Université Catholique du Graben, P.O. Box 29, Butembo, Democratic Republic of the Congo; 10 Department of Social and Preventive Medicine, School of Public Health, University of Montréal, PO Box 6128, Montréal, Canada; 11 School of Veterinary Sciences, The University of Queensland, P.O. Box 4072, Gatton, Australia

**Keywords:** *Taenia solium*, Epileptogenesis, Seizure, Neurocysticercosis, One Health

## Abstract

Neurocysticercosis (NCC), a central nervous system infection caused by the larval stage of *Taenia solium*, remains a leading cause of acquired epilepsy in endemic regions. Mounting evidence indicates that seizure development in NCC is not solely driven by host inflammatory responses but reflects complex, dynamic interactions between parasite development, host neuroimmune processes, and neuronal network remodeling. This narrative mini-review integrates experimental and clinical data to demonstrate that *T. solium* larvae actively shape the cerebral microenvironment through stage-specific immune modulation, blood–brain barrier disruption, and the release of neuroactive excretory–secretory products. During cyst degeneration, parasite antigens and excitatory amino acids drive microglial and astrocytic activation, amplify glutamatergic signaling, and destabilize inhibitory neurotransmission, collectively reducing seizure thresholds. These acute events are followed by chronic structural alterations, including perilesional gliosis, synaptic reorganization, and persistent network hyperexcitability, particularly around parenchymal and calcified lesions that serve as enduring epileptogenic foci. Integrating parasite developmental biology with neuroimmune and neuroexcitatory mechanisms, this review reconceptualizes NCC epileptogenesis as an active, multilevel dialogue between parasite, host, and neuron, rather than a passive, inflammation-driven consequence of infection. This integrated mechanistic framework highlights opportunities for biomarker discovery and therapeutic strategies that look beyond mere parasite eradication to address sustained neural dysfunction. It further underscores the critical need for One Health-oriented interventions to disrupt the *T. solium* transmission cycle and alleviate the long-term burden of NCC-associated epilepsy.

## Background

Neurocysticercosis (NCC), a parasitic infection of the central nervous system (CNS) caused by the larval stage of *Taenia solium*. affects millions globally, with the highest burden in low- and middle-income regions of Latin America, sub-Saharan Africa, and Asia [1, 2]. In these endemic settings, NCC accounts for up to one-third of epilepsy cases, establishing it as a predominant cause of acquired epilepsy worldwide [3]. Despite this significant impact, NCC is often underrecognized and inadequately managed, a problem compounded by limited access to neuroimaging, laboratory diagnostics, and specialized neurological care in resource-limited areas [4].

The clinical presentation of NCC is heterogeneous, ranging from asymptomatic infection to severe neurological disease manifesting as seizures, focal neurological deficits, hydrocephalus, and cognitive decline [5]. Seizures are the most frequent and often the earliest manifestation, occurring either acutely during cyst degeneration or evolving into chronic epilepsy due to persistent epileptogenic alterations in affected brain regions [6, 7]. However, the precise biological pathways linking *T. solium* infection to seizure initiation, propagation, and enduring network instability remain incompletely elucidated.

Increasing evidence indicates that epileptogenesis in NCC arises from dynamic interactions between parasite developmental stages and host neural and immune responses, rather than from inflammation alone. Parasite life cycle adaptations within the CNS, host inflammatory and neuroimmune signaling, direct neuroexcitatory effects of parasite-derived molecules, and progressive structural remodeling, including gliosis and calcification, shape seizure susceptibility and persistence [8–10]. While recent reviews have advanced our understanding of clinical manifestations and management [11], neuroimaging biomarkers [12], and broader parasitic contributions to epilepsy [13], these elements are often treated in isolation. This fragmented approach limits a cohesive mechanistic understanding of how parasite biology and host neural pathology converge to drive epileptogenesis.

In this narrative mini-review, we synthesize current evidence to frame NCC epileptogenesis as an active, multilevel process intrinsically linked to *T. solium* development and ongoing host-parasite interactions within the CNS. By bridging parasite developmental biology with neuroimmune mechanisms and direct neuronal excitability modulation, we advance past inflammation-centric models to delineate the interconnected pathways that culminate in seizure generation and chronic epilepsy. This integrated perspective aligns with emerging translational goals to identify mechanism-based biomarkers and therapies, while reinforcing the imperative for sustained public health efforts to interrupt *T. solium* transmission and mitigate the global burden of NCC-associated epilepsy.

## Life cycle of *T. solium* and neuro-invasion

The life cycle of *T. solium* involves humans as the definitive hosts, harboring the adult tapeworm in the intestine, and pigs as the typical intermediate hosts, where cysticerci develop preferentially in striated muscles (e.g., tongue, masseter, and limb muscles) [14]. Humans become accidental intermediate hosts through fecal-oral contamination via tainted food, water, or poor hand hygiene, leading to cysticercosis, including its NCC [15].

Following ingestion of eggs by a human host, the oncosphere penetrates the intestinal mucosa, enters the circulation, and disseminates to various tissues, including the CNS [16]. Blood–brain barrier (BBB) penetration is hypothesized to involve transient disruption of endothelial tight junctions and interactions with adhesion molecules, although precise mechanisms remain under investigation [17, 18]. Within the CNS, larvae typically enter a vesicular stage characterized by minimal inflammation, facilitated by the secretion of immunomodulatory molecules like prostaglandin E2 and transforming growth factor-beta (TGF-β), and the shedding of tegumental antigens that aid in immune evasion [19–21].

Clinical symptoms, particularly seizures, most commonly emerge during cyst degeneration. This process exposes parasitic antigens, provoking a robust host inflammatory response that damages surrounding neural tissue [11, 22]. In concert with factors like cyst location, developmental stage, and host’s immune status, this cascade disrupts neuronal networks via mechanical pressure, gliosis, and synaptic reorganization. These changes form the direct substrate for epileptogenesis and influence subsequent seizure phenotypes and potential pharmacoresistance [15].

## Immune-driven neuroinflammation and seizure induction

**Fig. 1:**
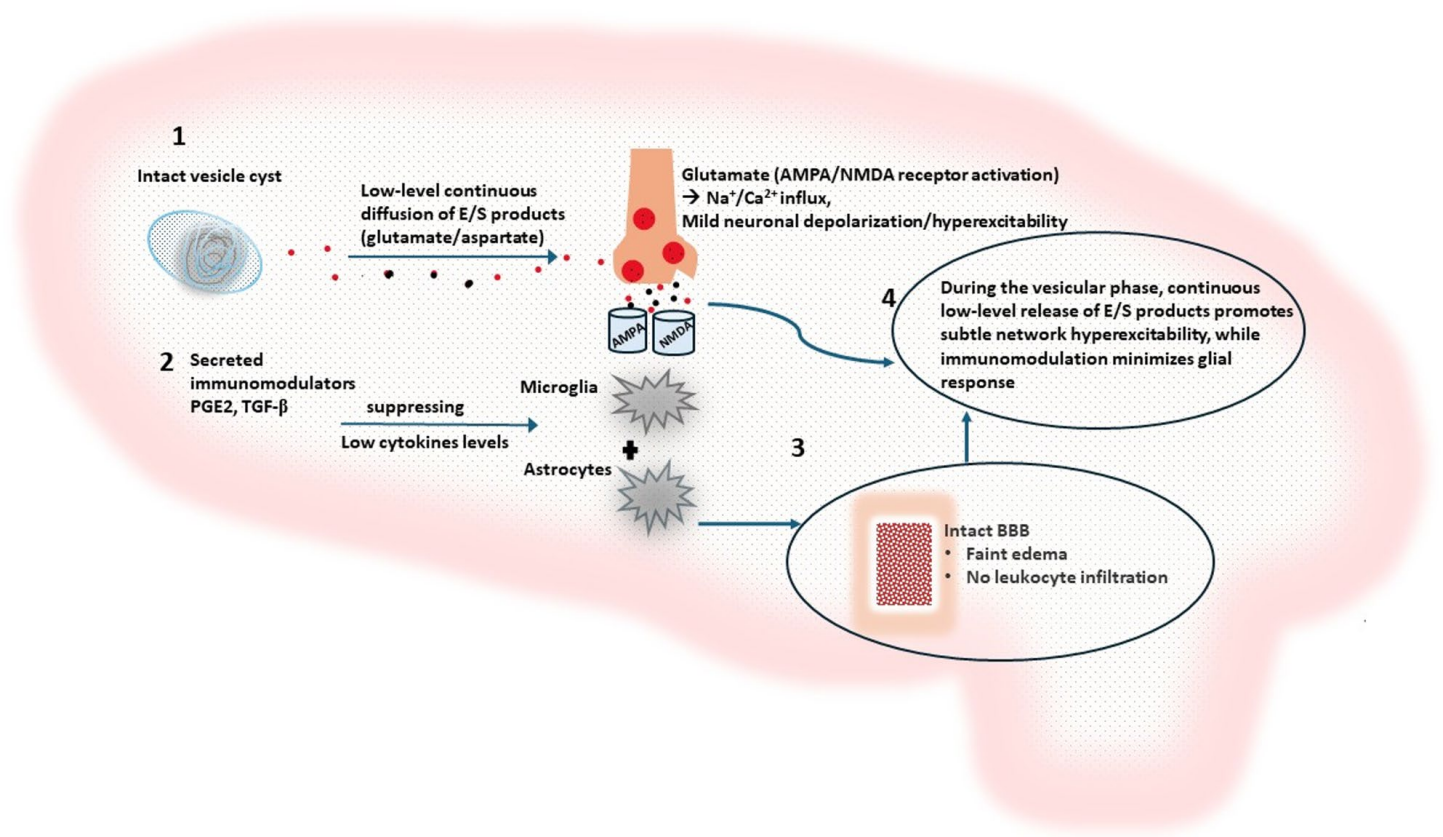
Immune-driven neuroinflammation and seizure induction in NCC

The host inflammatory response to degenerating cysticerci is a cornerstone of seizure genesis in NCC (Fig. 1). Exposure to parasitic antigens rapidly activates resident microglia and astrocytes, prompting a surge of pro-inflammatory cytokines, including interleukin-1β (IL-1β), tumor necrosis factor-α (TNF-α), and interferon-γ (IFN-γ) [23, 24]. Elevated levels of these mediators are consistently found in the cerebrospinal fluid and perilesional brain tissue of patients with active NCC [25]. Although counter-regulatory cytokines such as interleukin-10 (IL-10) and TGF-β are also upregulated, especially in later infection stages, this anti-inflammatory response often proves insufficient to quell the sustained pro-inflammatory signaling within epileptogenic niches.

These pro-inflammatory cytokines exacerbate blood–brain barrier (BBB) disruption, promoting vasogenic edema and facilitating the influx of peripheral immune cells like macrophages and lymphocytes [26]. The ensuing inflammatory milieu disrupts neuronal homeostasis by elevating extracellular glutamate concentrations while impairing GABAergic inhibition, effectively lowering the seizure threshold [27]. IL-1β plays a central role in this process by potentiating N-methyl-D-aspartate (NMDA) receptor activity, enhancing calcium influx, and amplifying neuronal excitability [28]. While regulatory cytokines may temper immune activation, they seldom reverse the synaptic dysfunction established by prior inflammatory excitation (Table 1).

**Table 1:** Mechanisms and molecular drivers of NCC-induced epilepsy

Mechanism	Core molecules	Supporting evidence	Clinical significance
Immune-driven neuroinflammation	IL-1β, TNF-α, IFN-γ, TGF-β	Microglial and astrocyte activation studies; rodent models of parasite antigen exposure	Lowers seizure threshold; contributes to acute and chronic seizures; correlates with perilesional edema and lesion load
Direct neuroexcitatory effects of parasitic products	Glutamate, Aspartate, other excretory-secretory (E/S) molecules	Hippocampal slice recordings; neuronal cultures exposed to *T. solium* E/S products	Induces epileptiform discharges independent of immune response; contributes to excitotoxic injury and network hyperexcitability
Blood-Brain Barrier disruption	Endothelial tight junction proteins (occluding, claudin-5)	Rodent NCC models; in vitro endothelial assays	Facilitates infiltration of immune cells and diffusion of neuroactive parasitic products, amplifying seizures
Astrocytic dysfunction and gliosis	GFAP upregulation, impaired glutamate uptake	Histological studies from human-resected tissue; rodent models of cyst degeneration	Impairs potassium buffering and glutamate clearance; promotes hyperexcitability and epileptogenesis
Structural and functional remodeling	Neuronal loss, synaptic reorganization, calcification	MRI and PET imaging; histopathology of cortical/subcortical lesions	Chronic seizure recurrence, pharmaco-resistance, and cognitive impairment; lesion location influences seizure propensity
Parasite antigen persistence	Ts8B2, other cyst-derived antigens	Serum and CSF antigen assays; rodent models	Continuous immune stimulation may trigger recurrent seizures around calcified lesions
Therapeutic modulation	Albendazole, Praziquantel, corticosteroids	Clinical trials, pediatric RCTs	Reduces parasite burden and antigen release; attenuates neuroinflammation; improves long-term seizure control

Astrocytic gliosis further exacerbates neuronal hyperexcitability by impairing potassium buffering and glutamate uptake, prolonging excitatory neurotransmission [29]. These alterations not only facilitate acute epileptiform activity but also promote enduring synaptic reorganization, cementing the epileptogenesis process [30]. Notably, calcified lesions once dismissed as inactive scars are now recognized as common sites of intermittent perilesional edema and recurrent seizures. This suggests that a smoldering, low-grade inflammatory process and incomplete immune resolution persist, continuing to drive epileptic activity long after the parasite itself has degenerated [31].

Degenerating cysticerci release parasitic antigens that activate microglia, resulting in the production of pro-inflammatory cytokines (IL-1β, TNF-α, IFN-γ). These signals drive BBB disruption with immune-cell infiltration and vasogenic edema. In parallel, astrocytes undergo reactive gliosis with impaired potassium buffering and glutamate clearance. Combined with cytokine-mediated calcium dysregulation, these alterations enhance neuronal excitability, promoting epileptiform activity that may persist within perilesional tissue surrounding calcified lesions.

## Direct neuro-excitatory effects of parasitic products

**Fig. 2:**
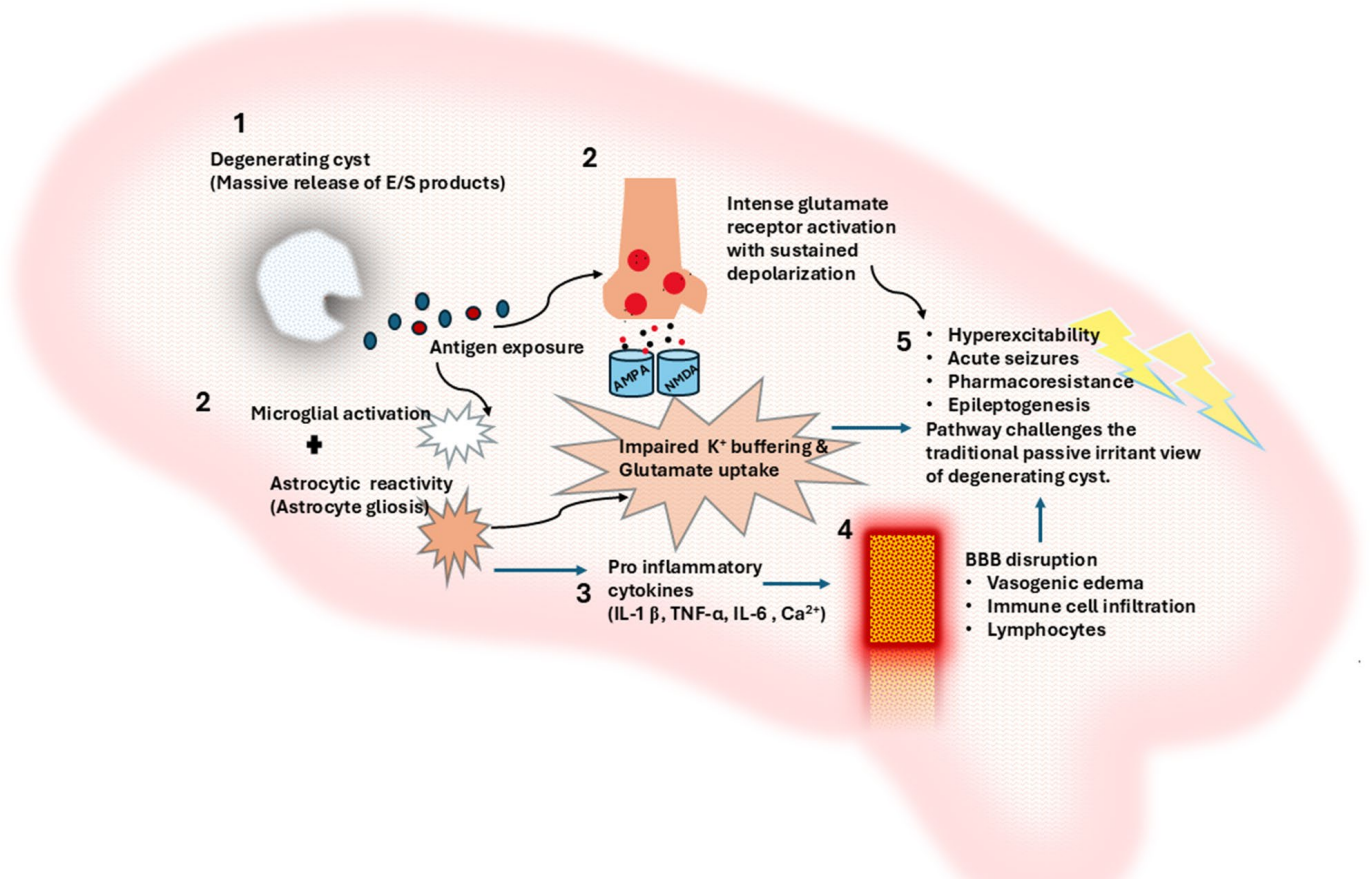
Direct neuroexcitatory effects of *T. solium* cysticerci

Beyond immune-mediated mechanisms, *T. solium* larvae can directly and indirectly modulate neuronal excitability through the secretion of neuroactive substances and interactions with resident glial cells (Fig. 2). In vitro studies using rodent hippocampal organotypic slices and acute human cortical brain slices have demonstrated that excretory-secretory (E/S) products from *Taenia* larvae can induce epileptiform activity via glutamate by receptors activation, pointing to a direct excitatory role of parasite‐derived glutamate on neuronal circuits (Table 1) [10].

These E/S products are rich in excitatory amino acids like glutamate and aspartate. The parasite likely expresses specific excitatory amino acid transporters, actively releasing these neurotransmitters into the immediate neural environment to drive sustained neuronal depolarization and network hyperexcitability [32]. During the relatively quiescent vesicular stage, larvae secrete immunomodulatory factors such as prostaglandin E2 and TGF-β, which dampen antigen presentation and curb microglial/astrocytic activation, maintaining an anti-inflammatory state.

However, upon cyst degeneration, stored E/S products are released into the parenchyma, and the concurrent disruption of the BBB facilitates their diffusion into surrounding tissue. Degeneration also exposes parasitic antigens that engage glial pattern recognition receptors, including Toll-like receptors (TLR2, TLR4) and NOD-like receptors, leading to microglial activation, astrocytic reactivity, and the release of pro-inflammatory cytokines such as IL-1β, TNF-α, and IL-6 [33–35]. The combination of glial-mediated inflammation, excitotoxic amino acids, and mechanical disruption of neuronal circuits contributes to network hyperexcitability, seizure generation, and pharmacoresistance. This dual-stage dynamic immune evasion during the vesicular persistence followed by a combined biochemical and immunological “attack” during degeneration challenges the notion of cysticerci as mere passive irritants, highlighting an active, multi-pronged pathway to epileptogenesis in NCC Table 1 integrates both immune-driven and direct neuroexcitatory pathways contributing to network hyperexcitability in NCC.

Degenerating cysticerci release excitatory amino acids that diffuse into adjacent brain tissue through localized BBB disruption. These factors activate neuronal AMPA and NMDA receptors, increasing synaptic excitation and promoting neuronal hyperexcitability independent of immune-mediated inflammation.

## Structural and functional brain changes underlying epileptogenesis

**Fig. 3:**
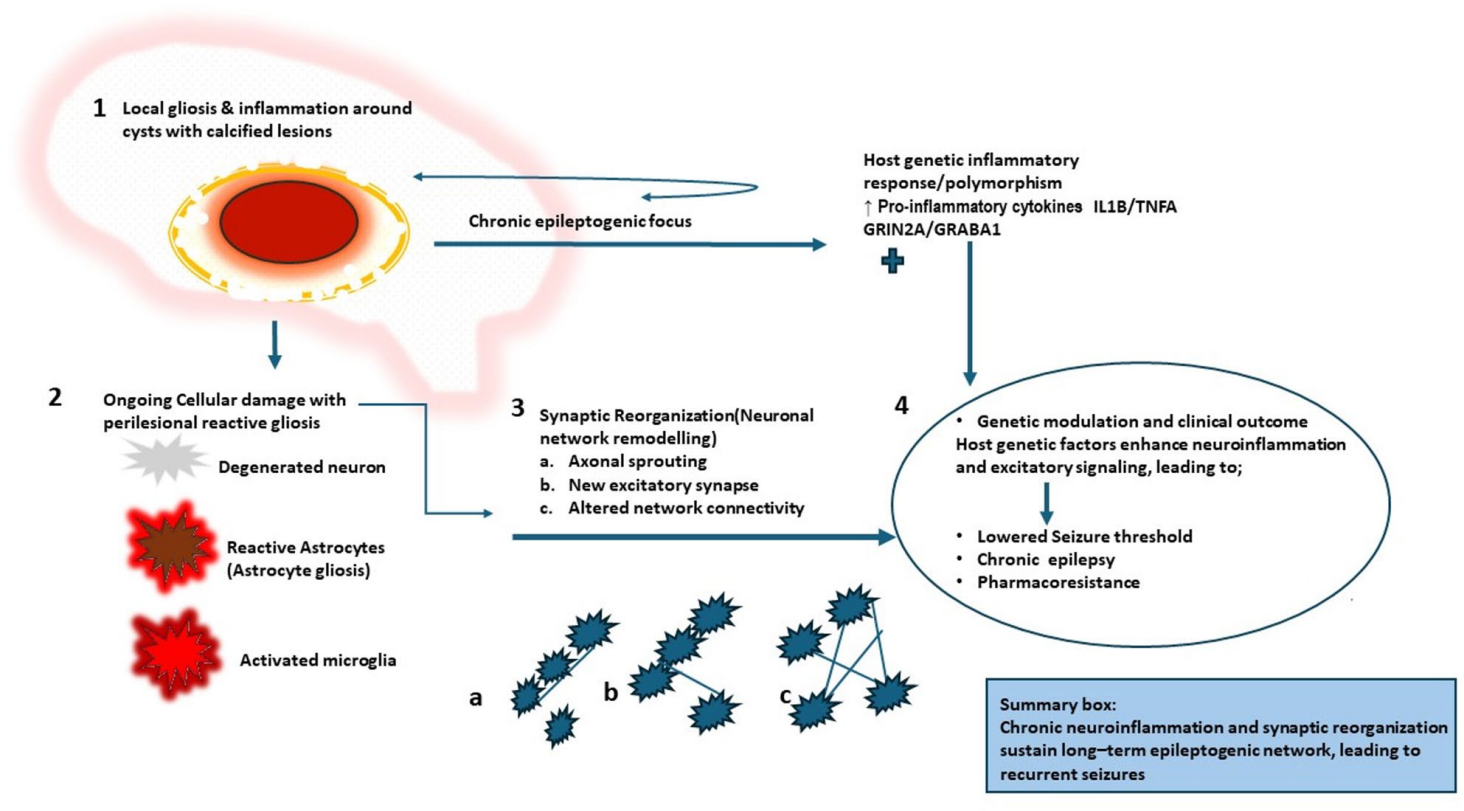
Structural and functional brain changes driving epileptogenesis in NCC

Epileptogenesis in NCC is underpinned by lasting structural, functional, and molecular remodeling of the brain (Fig. 3). Histopathological studies consistently show neuronal loss, reactive gliosis, and aberrant synaptic rewiring in the tissue enveloping cysts, creating a substrate for recurrent hyperexcitable circuits. Synaptic plasticity phenomena, such as axonal sprouting and the formation of new excitatory synapses, are particularly pronounced around parenchymal and cortical cysts, as well as calcified lesions. Here, perilesional gliosis establishes a chronic epileptogenic focus. These structural alterations, amplified by the local inflammatory environment generated during cyst degeneration, resemble patterns observed in other acquired epilepsies but are uniquely modulated by the presence of parasite antigens [35]. Structural remodelling and astrocytic dysfunction associated with chronic epileptogenesis are summarized in Table 1.

Neuroimaging corroborates these findings, revealing region-specific alterations in perfusion, metabolism, and functional connectivity in areas adjacent to cysts and calcifications. Such changes frequently correlate with seizure recurrence and pharmacoresistance [12, 36, 37]. Lesion location is a critical determinant of epileptogenicity: cortical regions (especially frontal, temporal, and parietal lobes) and subcortical white matter are highly susceptible to generating recurrent seizures, whereas ventricular or subarachnoid cysts tend to be less epileptogenic. Moreover, the presence of multiple cysts and a higher cumulative inflammatory burden significantly increase seizure risk and may contribute to pharmacoresistance [38–40]. Chronic gliosis around calcified cysts sustains hyperexcitable networks, forming long-term seizure foci. Mechanisms linking calcification, persistent antigen exposure, and seizure recurrence are detailed in Table 1.

Host genetic predisposition likely modulates these processes by influencing both inflammatory vigor and neuronal excitability. Polymorphisms in immune-regulatory genes (e.g., *IL1B* and *TNFA*) can amplify and prolong pro-inflammatory cytokine release during cyst degeneration, lowering seizure thresholds [41, 42]. Similarly, variants in genes governing excitatory and inhibitory neurotransmission, such as glutamatergic NMDA receptor subunits (*GRIN2A*) and GABAA receptor components (*GABRA1*), have been linked to altered cortical excitability and seizure susceptibility in focal and acquired epilepsies [43, 44]. Although direct genetic studies in NCC populations are sparse, these insights support a model where host genetics interact with parasite-induced inflammation, synaptic remodeling, and excitotoxicity to ultimately determine epileptogenicity, seizure recurrence patterns, and long-term treatment responses.

Parenchymal or cortical cysts incite perilesional gliosis and neuronal injury. Cellular damage, including degenerated neurons, reactive astrocytes, and activated microglia, promotes synaptic reorganization and altered network connectivity, leading to region-specific functional remodelling. Host genetic factors (*IL1B*, *TNFA*, *GRIN2A*, *GABRA1*) modulate the inflammtory and excitability landscape, collectively propelling the development of chronic, often drug-resistant epilepsy. Arrows depict the proposed progression from initial cyst formation to recurrent, drug-resistant seizures.

## Discussion

### Integrated mechanisms of seizure generation in NCC

Seizure generation in NCC epitomizes a complex synergy between parasite-derived neuroexcitatory forces and host-mediated neuroinflammatory cascades. During the degeneration of *T. solium* cysticerci, the unmasking of parasite antigens activates resident microglia and astrocytes, unleashing a sustained wave of pro-inflammatory cytokines like IL-1β and TNF-α. These cytokines recalibrate neuronal function by enhancing glutamatergic receptor activity, modifying ion channel dynamics, and increasing intracellular calcium influx, thereby priming networks for seizure initiation [45]. Simultaneously, the cysticerci discharge E/S products enriched in excitatory amino acids, including glutamate and aspartate, which can directly elicit epileptiform activity [10].

Neuroinflammation compounds this excitatory drive by crippling astrocytic glutamate clearance and potassium buffering, essential processes for maintaining neural homeostasis. Inflammation-induced BBB leakage further allows cytokines and parasite-derived neuroactive molecules to permeate surrounding cortical tissue [15, 16]. Together, these intertwined mechanisms forge a persistently hyperexcitable environment that not only triggers acute seizures but also lays the groundwork for long-term epileptogenesis, with especially detrimental consequences for the developing brain [10]. An integrated overview of these immune, excitotoxic, and structural pathways is provided in Table 1.

### Calcified lesions and epileptogenesis

The epileptogenic potential of calcified NCC lesions, despite being a common clinical observation, remains partially enigmatic. Once viewed as inert end-stage scars, calcifications are now frequently associated with recurrent seizures and episodic perilesional edema. Proposed explanations include intermittent immune reactivation by residual parasite antigens, persistent low-level microglial activation with cytokine release, and irreversible structural/gliotic scarring from prior inflammatory injury [40, 46]. This uncertainty is particularly consequential in pediatric populations, where early-life seizures can derail neurodevelopment and entrench epilepsy. Distinguishing between seizure driven by ongoing inflammation versus that arising from a fixed structural epileptogenic focus remains a major clinical challenge, hampered by a lack of reliable biomarkers. Bridging this diagnostic gap is essential for improving long-term neurological outcomes in children with NCC.

### Prevention and control under a One Health framework

NCC persists as a major driver of pediatric epilepsy and disability in sub-Saharan Africa (SSA). Effective combating NCC and its neurological sequelae demands strategies that transcend biomedical treatment, embracing a One Health approach that integrates human, animal, and environmental health. As highlighted by Sikakulya et al., the persistence of NCC in SSA exposes systemic deficits in healthcare access, veterinary services, sanitation infrastructure, and epilepsy surveillance, exacerbated by the limited involvement of neurologists in control programs. They contend that closing these gaps requires coordinated action targeting the entire parasite life cycle, not isolated measures [47].

This view is echoed by de Coster [48], who demonstrates that sustainable control of *T. solium* hinges on integrated tactics, including pig vaccination and anthelmintic treatment, improved meat inspection, community education, and environmental sanitation. Similarly, the World Health Organization advocates for linking neglected tropical disease programs with epilepsy surveillance, strengthening primary healthcare access, and systematically documenting infectious causes of epilepsy to guide policy and resource allocation [49].

Building on this, Sikakulya et al. explicitly frame NCC-associated epilepsy, especially in children, as a preventable neurological consequence of a neglected zoonosis [47]. Early engagement of neurologists, better documentation of infection-related epilepsy, and robust intersectoral collaboration can help intercept the neuroinflammatory and excitotoxic cascades that lead to chronic seizures. Collectively, these actions form the backbone of a viable One Health strategy to reduce the NCC burden and improve neurological outcomes for endemic pediatric populations.

### Biomarkers of NCC-related epilepsy

Biomarkers that capture inflammatory activity, excitotoxic stress, and parasite burden could enable earlier identification of individuals with NCC who are at high risk for epilepsy, particularly children. Cerebrospinal fluid (CSF) levels of IL-1β and TNF-α correlate with active disease and seizure occurrence, reflecting cytokine-mediated neuronal hyperexcitability [21]. Elevated glutamate in CSF or perilesional tissue also associates with seizure frequency and neuronal injury, offering a window into excitotoxic mechanisms, though it lacks disease specificity.

Parasite-specific antigens like Ts8B2 provide greater etiological specificity, mirroring ongoing parasite activity and antigen exposure (Table 1). Detectable Ts8B2 in serum or CSF could aid in monitoring disease activity and treatment response. A multimodal approach combining inflammatory, excitotoxic, and parasite-derived markers with neuroimaging features (e.g., lesion characteristics, perilesional edema) holds promise for enhancing diagnostic precision and stratifying seizure risk in endemic pediatric cohorts [12, 21].

### Therapeutic implications beyond parasite clearance

While albendazole and praziquantel are cornerstones of NCC therapy, their impact on epilepsy extends beyond simple parasite killing. Antiparasitic therapy reduces the antigenic load, thereby indirectly attenuating the chronic neuroinflammation that drives epileptogenesis-especially when co-administered with corticosteroids. This anti-inflammatory effect is crucial in children, where prolonged CNS inflammation can have lasting neurodevelopmental repercussions [46].

Successful cyst clearance also halts the continuous seepage of E/S products that contribute to glutamatergic overstimulation and excitotoxic damage. Although treatment may cause a transient inflammation flare during cyst degeneration, long-term outcomes typically include better seizure control. Pediatric studies suggest that combination therapy (albendazole plus praziquantel) achieves superior cysticidal efficacy and lesion resolution compared to albendazole monotherapy, which may reduce long-term epileptogenic potential [50, 51]. A deeper understanding of how antiparasitic drugs intersect with neuroimmune and excitatory pathways will be key to optimizing treatment regimens for pediatric NCC-associated epilepsy. Relevant therapeutic targets and pathways are outlined in Table 1.

### Review limitations

This review focused on parenchymal NCC, which constitutes the majority of epilepsy-related cases. Consequently, mechanisms specific to rarer forms such as intraventricular or subarachnoid disease are not covered in depth. Additionally, emerging therapeutic approaches targeting inflammation-driven epileptogenesis and the long-term neurodevelopmental consequences of childhood NCC fall outside our present scope.

Furthermore, the mechanistic understanding and public health response are constrained by limited surveillance data and systematic underreporting of infectious epilepsy etiologies, particularly in sub-Saharan Africa. Future research integrating neurological expertise, longitudinal patient cohorts, and One Health-informed surveillance systems are vital to address these gaps.

## Conclusions

NCC stands as a major cause of epilepsy in endemic regions, with seizure emerging from intricate crosstalk between *T. solium*, host immunity, and neural circuitry. Although inflammatory responses to cyst degeneration are central, compelling evidence now shows that parasite life cycle strategies and secreted molecules directly modulate neuronal excitability and network stability. These effects are compounded by chronic structural and inflammatory changes in the brain.

By weaving together parasite biology with neuroimmune and neuroexcitatory mechanisms, this review advances a model of NCC epileptogenesis as a biologically active, multi-layered process. This refined framework opens new avenues for biomarker discovery and therapeutic innovation aimed at both eliminating the parasite and ameliorating its neurological sequelae. It also reinforces the indispensable role of sustained public health initiatives to break the transmission cycle of *T. solium* and alleviate the global burden of NCC-associated epilepsy.

## Data Availability

Not applicable.

## Abbreviations

AMPA: α-amino-3-hydroxy-5-methyl-4-isoxazolepropionic acid
ASTMH: American Society of Tropical Medicine and Hygiene
BBB: Blood–brain barrier
CNS: Central nervous system
CSF: Cerebrospinal fluid
E/S: Excretory/secretory products
EEG: Electroencephalogram
GABA: Gamma-aminobutyric acid
IDSA: Infectious Diseases Society of America
IFN-γ: Interferon gamma
IL-1β: Interleukin-1 beta
MRI: Magnetic resonance imaging
NCC: Neurocysticercosis
NMDA: N-methyl-D-aspartate receptor
PET: Positron emission tomography
TNF-α: Tumor necrosis factor alpha
WHO: World Health Organization
